# Theories of Aging: An Ever-Evolving Field

**Published:** 2015

**Authors:** P. V. Sergiev, O. A. Dontsova, G. V. Berezkin

**Affiliations:** Belozersky Institute of Physico-Chemical Biology, Moscow State University, Leninskie Gory 1, bld. 40, 119992, Moscow, Russia; Chemistry Department, Moscow State University, Leninskie Gory, 1, bld. 3, 119992, Moscow, Russia; ESN group, Rochdel’skaya Str., 11/5, bld. 2, 123100, Moscow, Russia

**Keywords:** aging, life expectancy, reactive oxygen species, accumulation of damage, telomerase, advanced glycation end-product

## Abstract

Senescence has been the focus of research for many centuries. Despite significant progress in extending average human life expectancy, the process of aging remains largely elusive and, unfortunately, inevitable. In this review, we attempted to summarize the current theories of aging and the approaches to understanding it.

## INTRODUCTION

A number of theories, which fall into two main categories, have been proposed in an attempt to explain the process of aging. The first category is comprised of concepts holding that aging is programmed and those positing that aging is caused by the accumulation of damage. Conversely, the latter category of theories suggests various sources and targets of the damage. They are not necessarily mutually exclusive. Rather, aging could vary across different species, and programmed senescence can accelerate the buildup of damage or decrease the capacity for repair. What kinds of damage occur during aging?

## MITOCHONDRIA AND REACTIVE OXYGEN SPECIES

**Fig. 1:**
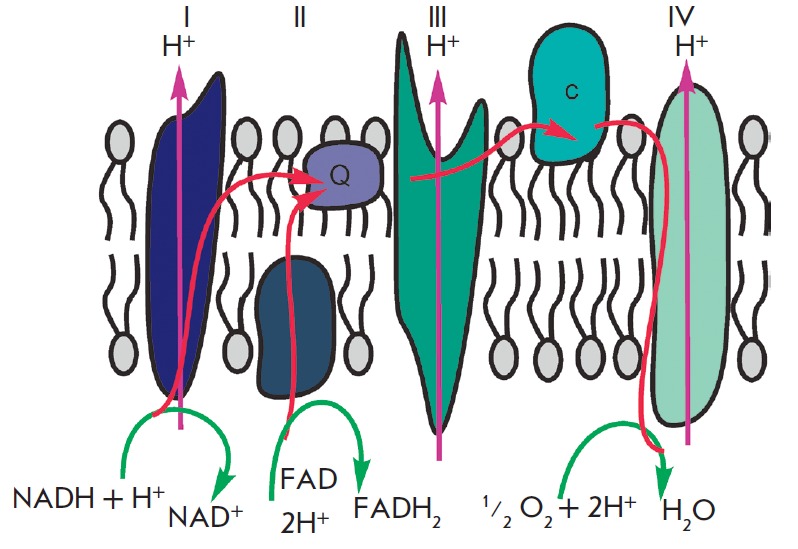
The mitochondrial respiratory chain, illustrating electron transfer from NADH and FADH_2_ to oxygen

**Fig. 2:**
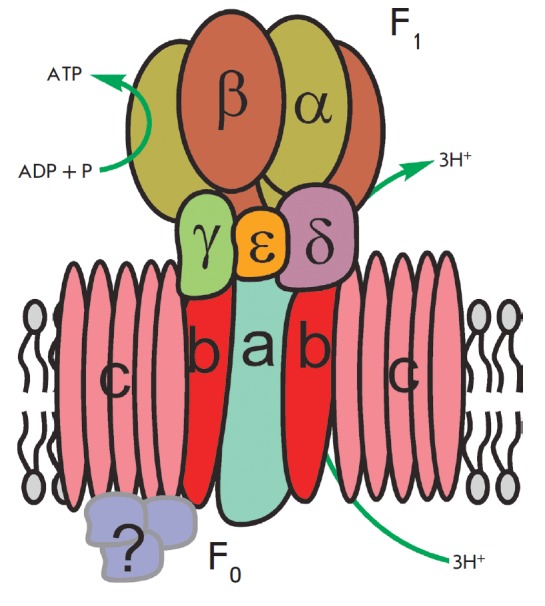
A schematic representation of ATP synthase structure and function

The primary function of mitochondria is respiration, which promotes energy production. Mitochondria break down organic compounds into water and carbon dioxide to release energy in the form of adenosine triphosphate (ATP). Each mitochondrion is contained in a double membrane. The outer membrane is relatively permeable to small molecules via transport proteins known as porins. The inner membrane forms folds (cristae) that increase the membrane area. Mitochondrial respiration generates a proton gradient across the inner membrane and a transmembrane potential through respiratory chain complexes (I–IV), enabling electron flow from the reduction equivalents NADH and FADH_2_ to oxygen. Simultaneously, the energy released in the oxidation of NADH and FADH_2_ is used to pump H+ ions out of the matrix into the space between the outer and inner membranes (*Fig. 1*). Thus, the intermembrane space of mitochondria is charged positively; and the matrix, negatively. Stored energy is used for ATP synthesis by the other membrane-bound protein complex – ATP synthase (*Fig. 2*).

A distinctive challenge for respiration is the release of excessive energy during the oxidation of organic molecules by oxygen (converted into the reduction equivalents NADH and FADH_2_). In this context, the respiratory chain is used to break the entire reaction into intermediate stages, the energy of which would be more efficiently saved (to establish a proton gradient). In addition, electrons could be transported one at a time or in pairs (as two reduction equivalents) in the respiratory chain. At the end of the catalytic cycle of oxygen reduction to two water molecules, four electrons are sequentially donated by reduced cytochrome *c *to cytochrome* c *oxidase.

During respiration, oxygen is reduced in several stages, producing a superoxide radical (O_2_^-^) and hydrogen peroxide. Most commonly, these molecules, known as reactive oxygen species (ROS), remain bound to cytochrome* c *oxidase until the reduction of oxygen to water is completed. In contrast to the common sequence of oxygen reduction by cytochrome *c *oxidase, oxygen molecules can occasionally form superoxide species by reacting with the reduced components of the electron transport chain. This typically occurs at the level of complexes I and III in the respiratory chain. In addition, the p66Shc protein can generate ROS via cytochrome *c* [1, 2]. Short-lived ROS are potent inducers of oxidative damage to any biomolecule. In particular, mitochondrially produced ROS inflict detrimental mutations on mtDNA. Mitochondria carry their own genome inherited from a bacterial ancestor living within early eukaryotes. mtDNA encodes mainly the RNA molecules needed for the synthesis of mitochondrial proteins and subunits of respiratory chain enzymes. Human mtDNA codes for two ribosomal RNAs and 22 transport RNAs, seven proteins of respiratory complex I (ND1, ND2, ND3, ND4, ND4L, ND5, ND6), one protein of respiratory complex III (CYB), three proteins of respiratory complex IV (CO1, CO_2_, CO3), and two protein subunits of ATP synthase (ATP6, ATP8). The majority of the other proteins essential for mitochondrial function are encoded by the nuclear genome.

Mutations in mitochondrial DNA can affect longevity. The most dramatic example is deficiency in the* COX5 *gene coding for the fifth subunit of cytochrome* c *oxidase in the fungus *Podospora anserina *[3], manifesting as a tenfold increase in the lifespan. Given that, impairment of the normal respiratory pathway in* P. anserine *leads to the use of the alternative pathway, which is private only in a handful of taxonomic groups.

Cells possess their own ROS metabolizing enzymes. The superoxide radical is converted by superoxide dismutase (SOD) to a less reactive hydrogen peroxide. Human cells harbor mitochondrial manganese superoxide dismutase (MnSOD) and few cellular copper-zinc superoxide dismutases. Hydrogen peroxide, which is produced from the superoxide radical or in other pathways, is broken down by catalase (CAT), peroxiredoxin (Prx), and glutathione peroxidase (GPx). Hydrogen peroxide can spontaneously react with ferrous ion (II) by the Fenton reaction to yield highly reactive hydroxyl radicals (OH•E), which can be detrimental to cellular functioning.

Viewing ROS as primary damaging molecules not only for mitochondria, but also for other cell compartments in cell senescence was proposed by D. Harman in 1956 [4] and has remained entrenched to this day. A decline in ROS production using mitochondria-targeted rechargeable antioxidants provided the basis for the approach proposed by V.P Skulachev to prevent age-related disorders [5]. Importantly, the role of ROS in age-associated pathologies has undergone several revisions in the past years. Originally, it was believed that damaged mitochondria increase ROS generation and thus accelerate aging [6]. However, it was eventually proved that most mitochondrial deficiencies do not end up with an elevated ROS but completely inactivated mitochondria, which led to the hypothesis that cells lacking a mitochondrial function pose a threat to the entire organism [7].

A few studies have challenged the negative role assigned to ROS as the primary mediators of cell damage in aging. Endogenously produced ROS in a wide range of animal species inversely correlate with lifespan [8]; however, experimental evidence suggests that the naked mole-rats *Heterocephalus glaber*, which demonstrate exceptional longevity, tolerate much higher levels of ROS and oxidative damage with regard to short-lived mice (*Mus musculus*) [9]. ROS are known to play an essential role in immune functioning, cellular signaling, and stress response (as reviewed in [10]). Special attention should be focused on the relationship between the ROS inactivation pathway and aging. Although it might appear that stronger protection against ROS extends the lifespan, in fact it does not. Conversely, a negative correlation has been established between the level of ROS metabolizing enzymes and longevity in mammals [8]. At the same time, exposure to the elevated enzyme concentrations involved in ROS inactivation results in lifespan extension. A positive impact was observed through genetic upregulation of catalase in mitochondria: not in the nuclei of murine cells [11]. Overexpression of CuZnSOD has been shown to increase the lifespan in adult *Drosophila melanogaster*. [12]. On the other hand, deletions in the genes implicated in ROS metabolizing pathways had no effect on the lifespan of the nematode *Caenorhabditis elegans*, while the deletion of the *sod-2 *gene even extended it [13].

Overall, ROS seem to play a detrimental role in cellular functioning, in particular mitochondria, during aging, but yet a beneficial role in other pathways.

## ACCUMULATION OF UNDEGRADABLE BY-PRODUCTS OF METABOLISM

**Fig. 3:**
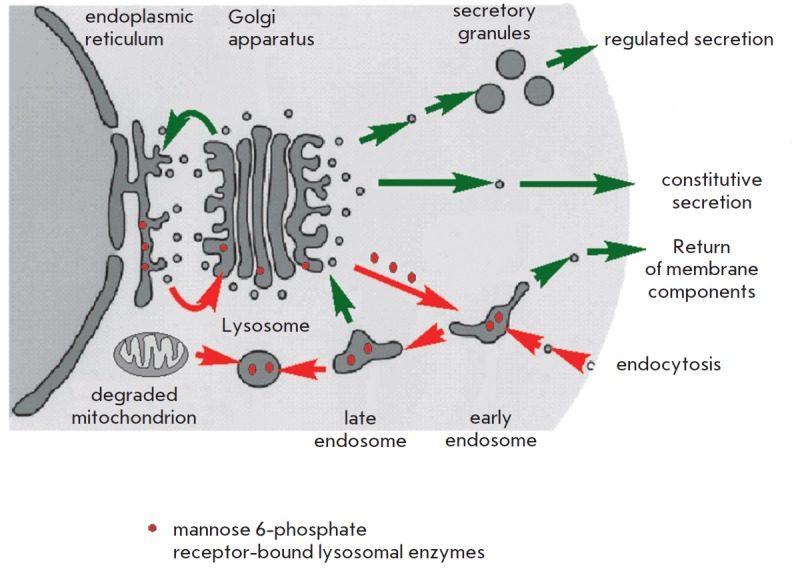
Lysosomal transport. A schematic representation of vesicular transport in the cell. Red arrows indicate the flow of material form the cellular membrane (material absorbed by the cell), the cytoplasm (for example, defective mitochondria to be degraded), and from the endoplasmic reticulum (lysosomal enzymes) to lysosomes

Another theory that attempts to explain the process of aging suggests that the accumulation of biological garbage that cannot be completely removed from the organism is responsible for cell senescence. In its basal form, this theory was described by V. Gladyshev [14, 15]. Its aspects were presented in detail in [16]. It holds that, due to the stochastic nature of biochemical reactions, including enzymatic pathways, side reactions have the potential to occur. The degree of complexity of a biochemical network contributes to the range of by-products formed. Some of them are readily eliminated by excretion or degradation. Each by-product is broken down by an appropriate enzyme or a series of enzymes, which in turns makes the metabolism more complex and increases the array of by-products. Enzymatic pathways for metabolizing by-products vary across species. These pathways, though not numerous, tend to be restricted to only the most commonly produced compounds with toxic properties, allowing other by-products to build up. The only mechanism by which these agents are diluted in the cells is cell division. This only applies to replicative cells. The challenge for multicellular organisms such as the human organism is that many cell types lose replicative capacity or divide slowly, even though they remain active throughout the lifespan. These cells, including cardiomyocytes and brain neurons, accumulate metabolic waste that eventually affects normal cell functioning. A common by-product of cellular metabolism seems to be lipofuscin, a substance composed of non-degradable material that accumulates in lysosomes. Lysosomes are intracellular organelles serving as degradative compartments for intra- and extracellular components. In addition, the digestion enzyme hydrolases contained in lysosomes are transported in vesicles from the endoplasmic reticulum and the Golgi apparatus. Hydrolases are transferred to lysosomes after binding to mannose 6-phosphate residues (*Fig. 3*). Lysosomal enzymes are active at acidic lysosomal pH only. Lipofuscin deposition decreases enzyme levels and impairs lysosomal acidification, which ultimately affects hydrolase activity. Each organism displays species-specific groups of lysosomal enzymes and the enzymes responsible for the breakdown of metabolic debris in other cellular compartments.

**Fig. 4:**
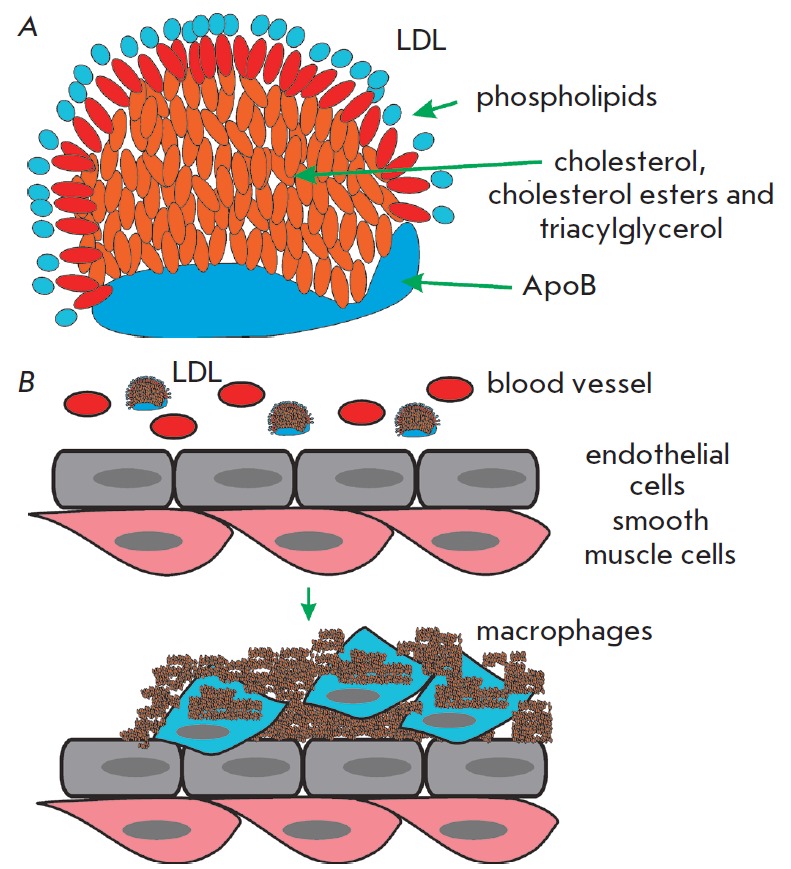
Structure of the low-density lipoprotein (LDL), the major carrier of plasma lipids (*A*), and formation of the atherosclerotic plaque (*B*)

The accumulation of non-degradable material can occur in the intra- and extracellular environments. Among the extracellular deposits found in humans, cholesterol-containing plaques and their oxidized derivatives in blood vessels are worth mentioning, as well as protein polymers, such as β-amyloid in the central nervous system. Atherosclerotic plaques contain lipids deposited in the walls of blood vessels. Firstly, there is oxidized and glycated cholesterol derivatives; however, other lipids may be present. Low-density lipoproteins (LDL) transport fat molecules around the body, from where they accumulate on the walls of arteries (*Fig. 4A*).

Atherosclerotic plaques serve as a site for the recruitment of monocytes, which eventually differentiate into macrophages. These immune cells absorb cholesterol and prove beneficial at some point. However, under certain conditions, macrophages accumulate in plaques and form lipid-loaded foam cells (*Fig. 4B*). A comparison of DNA polymorphisms in French centenarians with control individuals in a genome*-*wide association study (GWAS) demonstrated that one of the ApoE genotypes (E2 allele), a component of very low density lipoproteins, was significantly more frequent in the centenarian group, whereas the E4 allele, associated with a high risk of atherosclerosis, was significantly less frequently present [17]. Moreover, ApoB alleles, a major component of low density lipoproteins, had no association with longevity.

**Fig. 5:**
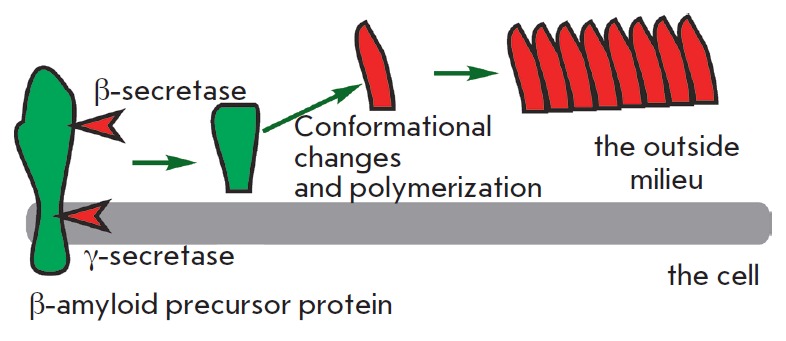
Formation of β -amyloid and polymerization

Amyloid proteins are another class of toxic waste accumulating mainly in the nervous system. The best-described amyloid protein is β-amyloid, which is known to cause Alzheimer’s disease (reviewed in [18]). It is generated from a functionally important protein, the amyloid precursor protein, via cleavage of the precursor molecule at both termini by β- and γ-secretases (*Fig. 5*). The β-amyloid protein can exist in several forms, one of which, rich in β-sheets, is toxic. The toxicity is due to β-amyloid polymerization, which can induce other monomers to accept the misfolded structure. Amyloid β-peptide polymerization results in amyloid plaque formation in nerve cells, causing Alzheimer’s disease. There is evidence suggesting that β-amyloid peptides can spontaneously undergo pyroglutamate modification and acquire a higher toxicity [19]. There is a wide array of compounds, other than β-amyloid peptides, capable of self-polymerization into toxic insoluble structures. It is likely that a spontaneous modification of proteins also plays a role in the formation of metabolic debris.

**Fig. 6:**
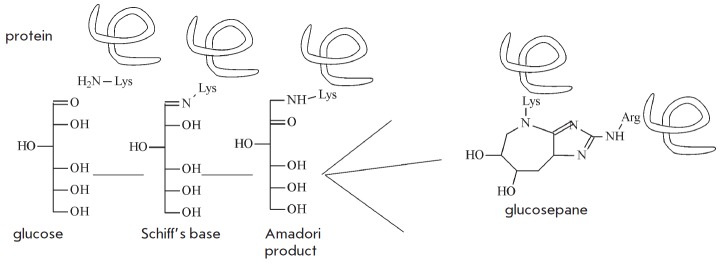
Spontaneous glycation of proteins

Finally, metabolic waste also includes, to a certain extent, spontaneously modified sugar-bound proteins, mainly glucose molecules. Glycation involves interaction between the amino groups of lysine and the aldehyde groups of glucose (*Fig. 6*) via a Schiff base reaction. It is followed by rearrangement of the double C=N-bond, known as Amadori products, to yield a wide range of advanced glycation end-products such as glucosepane.

The main consequence of spontaneous glycation is impaired elasticity, which is essential to blood vessels [20]. In addition, spontaneous glycation affects protein functioning. This process well describes the concept of accumulation of metabolic waste that promotes aging. Until now, no enzyme has been discovered that is capable of metabolizing glycated products. Preventing spontaneous glycation seems to be impossible, because all proteins contain lysine residues and glucose is one of the important substances in all living organisms. FAD-dependent deglycating enzymes have been found in fungi (amadoriase) and bacteria (fructoselysine-6-kinase frlD and fructoselysine -6-phospahte-deglycase frlB) [21], though they can only act on low-molecular weight molecules, such as amino acids conjugated to sugars, and have no activity towards glycated proteins. In vertebrates, fructosamine-3-kinase (FN3K) has been identified, together with a related protein (FN3K-RP), a breaker of glycation end products. It is an ATP-dependent enzyme that targets only intracellular, rather than extracellular, molecules.

## IMPAIRMENT OF REGULATORY PATHWAYS DURING AGING

Aging is associated not only with the buildup of metabolic by-products, but also with the dysregulation of regulatory pathways. For example, aging upsets the balance between pro- and anti-inflammatory components, promoting chronic inflammation. The causality between such inflammatory processes and age-related disorders has been stated in the inflamm-aging theory of Franceschi [22]. An elevated predisposition to inflammatory diseases in early age, as a protective barrier against infection, proves to be detrimental in the elderly.

Besides the imbalance in pro- and anti-inflammatory responsiveness, aging can also impair other important pathways. The Russian researcher V.M. Dilman conceptualized the neuroendocrinal theory (elevation hypothesis) of aging [23]. This theory involves the existence of self-regulatory mechanisms of homeostasis – negative feedback pathways. One of the essential systems is the hypothalamus-pituitary-adrenal axis. An elevation of the threshold of the hypothalamus to negative feedback signaling accounts for the unfavorable age-related changes in human health; in particular, reproductive decline [24]. The development and experimental verification of the elevation hypothesis represent an important achievement in aging research in Russia.

## TELOMERES ARE THE BIOLOGICAL CLOCKS OF THE CELL

Eukaryotic DNA is organized into linear, double- stranded chromosomes. The number of chromosomes varies from one to several hundred from species to species. Linear chromosomes are capped by repetitive nucleoprotein structures known as telomeres that protect the chromosome ends against degradation and fusion. Telomeres allow cells to distinguish the appropriate chromosome ends from the double-strand DNA breaks induced by exogenous factors like radiation. The linear arrangement is one cause of the end-replication problem first articulated by A.M. Olovnikov [25]. DNA replication requires an RNA primer to initiate synthesis, followed by its removal, which progressively shortens chromosome ends with each cell cycle. Olovnikov suggested the existence of an enzyme capable of elongating the ends. Long afterward, such an enzyme, called telomerase, was experimentally confirmed [26].

Telomerase activity was not shown in all cell types. Cells with unlimited proliferative potential such as germ and stem cells can extend telomeric DNA via telomerase. The majority of other cell types have a finite replicative capacity or are non-dividing. Such cells lack telomerase activity, thus suffering telomere attrition upon successive cell divisions [27]. This erosion provides an explanation for the observation of a limited lifespan in cultured somatic cells [28]. By and large, telomere shortening is a type of molecular clock that counts cell divisions. It is tempting to extrapolate this clock into the context of the entire organism, assuming that telomerase activation can confer replicative immortality to somatic (non-germ) cells. However, such a straightforward approach encounters serious challenges. The absence of telomerase activity in many cell types in a multicellular environment serves as a mechanism by which malignancy is suppressed [29]. Even in the event of mutations leading to uncontrolled growth regardless of cell-division pathways, such cells would have a finite lifespan anyway in the absence of telomerase activity. Many tumors carry mutations that upregulate telomerase and increase proliferative capacity in a small population of tumor cells with telomerase activity. Owing to this mechanism, malignancy rates are not high in contrast to the presence of telomerase in all cells. Overall, there is a trade-off between physiological cell turnover and the occurrence of tumor cells. An insufficient amount of cells with telomerase activity would lead to poor tissue renewal, whereas an elevated number of telomerase-positive cells would increase malignancy rates.

## BIOLOGICAL CLOCKS AND METABOLISM RATES

It has been long known that dietary restrictions prolong the lifespan in various organisms. This observation was made by McCay *et al*. through studies of mice in the 1930s [30]. Since then, a wide array of mutations have been identified in genes that affect metabolic changes, which proved to increase the lifespan in the model animals. Extensive work has been performed on the nematode *C. elegans*, a favorable system for studying developmental biology. This tiny organism has a fixed number of cells, with each cell’s fate predetermined. Some mutations lead to a twofold lifespan increase in *C. elegans *[31], while its normal life-span is 20 days. The mutations increasing the lifespan of *C. elegans *have been described in detail in [32]. Most of the mutations which positively affected the lifespan of the nematode had an effect on metabolic activity. Upon starvation, *C. elegans *can enter a state of dormancy.

This program, the dauer stage (enduring, persisting), involves a slowed metabolism and larval development, as well as decreased food intake, and of course reproductive arrest. In this state of dormancy, the worm can survive food deprivation by increasing its lifespan. Since the dauer state is naturally triggered by larval starvation, it is very similar to the lifespan extension in other organisms following dietary restrictions. The distinct difference is that highly organized organisms have no specific developmental strategies in the case of food deprivation.

**Fig. 7:**
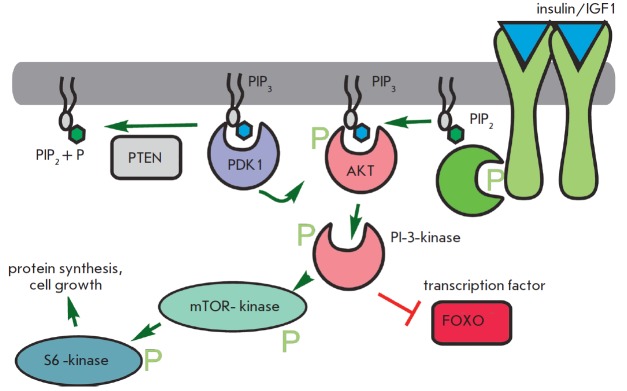
Simplistic representation of the insulin/IGF-1 signal pathway

The *daf-2 gene *mutation, which causes a two-fold lifespan increase in *C. elegans, *is related to the insulin receptor gene [33]. Other lifespan extension mutations also suggest a role for insulin-related pathways and the insulin-like growth factor (IGF1) (*Fig. 7*). These pathways, which are triggered by increased food intake, primarily glucose, elevate the metabolic rate, as well as promote growth and cell division. These mutations occur in genes encoding early and late components of the signaling cascade. Phosphoinosite- 3-kinase (age-1) transmits a signal from the insulin-like receptor substrate (IRS) PDK and Akt kinases, which in turn mediate signaling to protein synthesis and alter transcriptional regulation through the FOXO transcription factor (daf-16). Another important component controlling metabolism and aging is histone deacetylase Sir2. Histone deacetylation results in transcriptional repression. Upregulation of Sir2 increased longevity, even though with deletion of this gene calorie restriction has no greater impact on the lifespan [34].

A handful of genes of the fruit fly *Drosophila melanogaster* have been mapped and implicated in lifespan extension [32]. Genetic analysis has shown that a significant increase in the lifespan is associated with mutations in the signal transduction pathways from the insulin and insulin-like growth factors and with mutations directly affecting the metabolic activity and the Krebs cycle. Nearly the same spectrum of cellular regulatory pathways is altered with lifespan extension mutations in mice [32]. Among these longevity genes are genes encoding elements from insulin and insulin-like growth factor signaling and those encoding stress response proteins.

Overall, the analysis of the mutations that cause lifespan increase in various model organisms brings us closer to understanding the concept of biological clocks, which behave as a function of time and the metabolic rate. In complete agreement with the concept by V. Gladyshev and other theories of aging, metabolic activity mainly accounts for the formation of by-products that fail to undergo elimination and accumulate. A higher metabolic rate contributes to a faster buildup of toxic metabolic waste and lesions. Conversely, a lowered metabolism either in the context of calorie restriction or mutations affecting metabolic pathways, triggered by increased food intake, promotes lifespan extension through a decline in the accumulation of toxic by-products.

Mechanisms whereby lesions occur in normal metabolism are also related. First and foremost, it is the formation of reactive oxygen species that damage cellular components and glycation of the cellular components induced by glucose, an essential macronutrient for cells. Glycation and oxidation products contribute to lipofuscin formation in lysosomes, reduced vessel elasticity, and deposition of insoluble aggregates on the walls of blood vessels and the nerve tissue.

## IS SENESCENCE PROGRAMED?

Although many molecular mechanisms of aging have been studied and are akin to an inevitable accumulation of toxic metabolic waste products or damage caused by them, there have been established theories claiming that aging is programmed. The theory of programmed senescence was first described by A. Weismann [35]. Later on, V. Skulachev extended this theory, which received much attention [36]. There is another theory of programmed aging by A. Boiko [37]. The theories of programmed aging and spontaneous senescence are often shared by molecular mechanisms.

What are the arguments to support the programmed aging theory? First, some species senesce abruptly, which undoubtedly appears to be programmed. Rapid senescence is nearly always initiated following reproduction. Bamboo reproduces vegetatively and can grow for 15–20 years without apparent senescence. After flowering and seed formation, it rapidly withers away, thus allowing seeds to germinate. The most salient example of accelerated aging is the salmon. Salmon migrate from the Pacific to the rivers, where they spawn eggs, followed by a marked elevation in the plasma levels of glucose, fatty acids, cholesterol, and adrenal secretion and death. An example of avoiding entering the state of accelerated aging was discovered by the Russian biologist V.V. Zyuganov [38]. The pearl mussel larvae parasitizing in the gills of salmon delay senescence in salmon for them to mature. The hypothesis of induced delay of senescence in the salmon was criticized [39], even though dying a programmed death cannot be doubted. There are a few other examples of apparent programmed death that are frequently linked to the reproductive function, such as death following copulation in males of the brown antechinus [40] and mayflies or post-copulatory decapitation by female mantises. These features are a clear indication of programmed death; however, its links to aging are often obscured. Yet, these examples show a potential for life-history strategies deleterious for an individual but beneficial to the entire population. The theory of V. Skulachev [36] holds that senescence of mammals is a deleterious program of the kind, though spread over an extended period and implemented through the formation of ROS in mitochondria.

Most obviously, the average lifespan within a given species is genetically programmed in one way or the other. Nevertheless, the current theories of aging differ in viewing aging as a consequence or a side effect of genetic pathways. According to the well-known disposable soma theory suggested by T. Kirkwood [41], aging is a trade-off in the allocation of limited energy resources between maintenance and restoration of tissue homeostasis and other traits needed for survival. This trade-off is demonstrated when comparing the mean lifespan of related animal species with different predation risks. When the risk is high, delayed senescence has no added benefit relative to, for example, rapid reproduction. According to A. Boiko [37], senescence is an acquired program. Ancestors of multicellular animals and many present-day taxa of multicellular species are devoid of such a program. Aging in itself is genetically programmed in ontogenesis, involving the formation of non-renewable tissues – the so-called post-mitotic tissues. The cells of such tissues are non-dividing and cannot be renewed by stem cell populations. The theory of Boiko thus incorporates the theory of by-product accumulation by V. Gladyshev [15] and seems to be well articulated.

## COMPARATIVE GENETICS OF LONGEVITY

The aging program is explicitly or implicitly encoded in the genome and, in theory, could be captured by comparing the genomes of aging and non-aging organisms. However, organisms should be related to avoid much variation in aging-unrelated genes across their genomes. Even genome wide association studies of longlived and control individuals do not always provide unambiguous conclusions. There is a great body of work concerning a possible association between longevity and mutations (allelic polymorphisms); however, statistical significance has been a challenge. Current studies in this field involve several thousand DNA samples both from the control and long-lived individuals. Only a single gene, the *APOE *gene, has been statistically linked to longevity (*p * < 5 × 10^-8^) [42]. Among the associations with a lower statistical significance are elements of the insulin*/*IGF-1 signal pathway (AKT1, AKT3, FOXO4, IGF2, INS, PIK3CA, SGK, SGK2, YWHAG) and telomerase (POT1) [43]. Lifespan extension mutations have been recently reviewed by Newman [44].

Genome-wide analysis of long-lived individuals within a given species with controls allows one to identify the genes affecting longevity, but there are specific aspects. The benefit of this approach is that individuals recruited to such a study possess highly related genomes, which enables a differentiation between relevant and non-relevant mutations with a high statistical significance. The human genome of 3×10^9^ bp carries several million individual polymorphic sites [45]. Using such tools as microarrays, up to 1,000,000 loci could be analyzed per individual. Due to the high number of differences, statistical significance is set at *p * < 10^-8^. Yet, the drawback of such studies is the low variation in life expectancy. Genome analysis of different species could theoretically reveal the genes affecting the lifespan to a higher extent (several-fold). The lifespan of animals can vary up to 10, 000-fold. For example, rotifers live several days, and the great polar whale’s lifespan is up to 200 years. Genome analysis of species with various lifespans poses additional challenges. Even representatives of a single species carry millions of differences. The genomes of different species differ to a degree that makes their comparison, if at all possible, infeasible.

The lifespan potential can vary up to 10,000-fold only in organisms that dramatically differ in morphology and body size range. There is no point in attempting to discover genes affecting longevity between rotifers and the great polar whale. In general, the lifespan is significantly governed by body size [46].

A feasible approach to unraveling the genetic background behind longevity is to compare genetically related similar-sized species with various lifespans. Among small mammals, flying bats have a longer lifespan and marsupials have a shorter lifespan than expected. Birds and bats have a longer mean lifespan as compared to similar-sized terrestrial animals. This is likely due to lower susceptibility to predation. Early death disfavors selection for individuals with longlived genetic backgrounds, whereas the lack of predation risks favors selection for long-lived individuals.

There are a handful of mammals that are distinguishable as unusually long-lived species with shortlived counterparts. The best-known example is the naked mole rat *H. glaber *with a maximum lifespan of over 30 years, which is a 9-fold difference to the related mouse. The naked mole rat is a burrowing rodent native to Africa. It is the only truly eusocial mammal such as ants or bees. Each underground colony, which rodents never leave, has a reproductive queen that stops other females from breeding. The other naked mole rats, workers and soldiers, feed the queen and protect the colony against neighbor colonies or snakes – the main predator of naked mole-rats. The naked molerat is insensitive to pain and cold, tolerant to low-oxygen environments containing only 8% oxygen and 25% carbon dioxide. These rodents are known to show high resistance to cancer and their mortality rates do not increase with age without apparent aging. The genome of the naked mole-rat has been determined by the laboratory of V. Gladyshev [47] and comprises 22,561 genes, with 750 genes acquired and 320 genes lost during evolution. A total of 244 pseudogenes–non-functional genes–have been identified. Among the pseudogenes are gene clusters with homology to the genes involved in ribosomal and nucleotide synthesis pathways, olfactory and vision systems, spermatogenesis and, possibly, ubiquitination – ubiquitin tagging of proteins for degradation. The putative telomere or the telomerase genes *TEP1 *and *TERF1 *have been found to be unique to the genome of the naked mole-rat. Forty-five amino acid substitutions were found in 39 naked mole rat proteins, not occurring at the same positions in other mammals. These proteins include components of replication and DNA integrity systems: CCNE1, APEX1, RFC1, TOP2A. In addition, unique substitutions were detected in the genes of body temperature maintenance (UCP1) and vision (CRYGS). The genome of *H. glaber* contains 1.87 million polymorphic loci. The frequency of polymorphic variants is more similar to that observed in humans than in rats and mice related to the naked mole rat. The analysis of the expression profiles of 33 genes affected by aging in the human brain revealed that 32 of the corresponding genes of the naked molerat were not affected. Among these are the *CYP46A1* gene regulating cholesterol metabolism and amyloid plaque formation and the *SMAD3 *gene encoding a transcription factor that delays cell division and promotes tumor growth. The naked mole rat has impaired melatonin secretion, which, similarly to melatonin-deficient mice, is consistent with down-regulation of the insulin*/*IGF-1 signal pathway. A consequence of adaptation to oxygen deprivation seems to be mutations in the hypoxia-induced factor (HIF1α) and VHL, a protein regulating HIF1α. The genome of *H. glaber *is an interesting model for studying longevity genes.

Another long-lived mammal whose genome has been recently annotated by V. Gladyshev’s laboratory [48] is the Brandt’s bat. In view of the above, bats display an exceptional longevity relative to similar-sized mammals. The lifespan of the Brandt’s bat is over 40 years, which is the longest on record in the context of the positive relationship between longevity and body size, given the bat weight of 4–8 g. The genome of this nocturnal insectivorous mammal contains 22,256 genes and 194 pseudogenes, comprising 2 × 10^9^ nucleotides. A total of 67 gene families significantly expanded, and 44 gene families contracted. Immunity-related genes within the expanded gene families deserve a closer look. In the course of evolution, the Brandt’s bat acquired 349 genes and lost 98 genes. Some genes are involved in echolocation, visual adaptation to low light conditions, and hibernation. Putative lifespan extension mutations are detected in growth hormone receptors (GHR) and insulin-like growth factors (IGF1R). Mutations in the *IGF1R *gene (*daf-2*) have been found in long-lived mutants of *C. elegans*. The expression profiles of the indulin/IGF1 pathway, like *FOXO1, *in the Brandt’s bat were shown to change in a similar fashion to mutant long-lived mice and to be typical of a slowed metabolism.

## SEA URCHINS AS A MODEL FOR COMPARATIVE GENOMICS OF LONGEVITY

Sea urchins belong to the phylum Echinodermata, the superphyllum Deuterostomia as vertebrates. They are closer relatives to vertebrate animals than to such protostomes as arthropods and mollusks. Adult sea urchins possess a five-fold symmetry. Sea urchins are enclosed in a calcareous globe-shaped shell, consisting of rows of plates with a pentameric symmetry as well. The mouth is located on the underside and the anus, on the top of the body. The body is covered with flexible spines moved by species muscles. Sea urchins have received attention as a model for developmental biology. Sperm and oocytes are released into the sea water and could be produced under laboratory conditions. Fertilization occurs externally in water, followed by immediate cell division. These creatures stirred another wave of interest as long-lived individuals. *Strongylocentrotus franciscanus*, or the red sea urchin, is found in the Pacific Ocean along the North American coast in the cold California current. The exceptional longevity of this species has been confirmed. Tetracycline injected into the red urchin is deposited in the calcareous shell. One-year-post-injection collection and analysis of sea urchins allows one to evaluate annual growth bands. It was shown that a period of accelerated growth is followed by a pronounced slowdown [49].

A statistical analysis of variation in the body size of* S. franciscanus *made it possible to estimate the maximum lifespan. Larger sized individuals can survive into old age, exceeding at least 100 years. Another confirmation was obtained with radiocarbon distribution (^14^C) in the calcareous teeth of *S. franciscanus*. An enhanced amount of radiocarbon in the world ocean, due to nuclear-bomb testing in the 1950s, was used as a marker for evaluating the mean tooth growth in the red sea urchin for a period of over several decades [50]. Both studies [49, 50] demonstrated that *S. franciscanus* lives over 100 years. Importantly, only few species of sea urchins show an extended longevity. Another sea urchin found in the Pacific Ocean, *S. purpuratus, *sharing the habitat with *S. franciscanus, *displays a long lifespan of 50 years, but not so much as that seen in the red seas urchin. At the same time, the variegated sea urchin, *Lytechinus variegates, *lives only 3–4 years [51]. The dramatic disparity in longevity among related species holds prospect in using these animals as models for gaining insight into the genetic background of longevity.

## CONCLUSIONS

Nearly all current theories of aging have in common the fact that the fundamental cause of aging is the accumulation of molecular damage brought about mainly by ROS, but the role of amyloid protein, glycation end-products, and lipofuscin is acknowledged as well. The current theories differ in the extent to which the buildup of waste is encoded in the genome and whether it is programmed death or this accumulation that is deemed to bear the costs of evolutionary benefits. In addition to damage itself, the rate of accumulation is also of concern, which results from overall metabolic activity. The most significant changes in the longevity of model organisms prove to be mutations in metabolic pathways. Alongside the analysis of model organisms, it is possible to extend to a genome-wide analysis of longlived animals and short-lived counterpart species.
